# The Influence of Taurine Supplementation on Serum and Tissular Fe, Zn and Cu Levels in Normal and Diet-Induced Insulin-Resistant Rats

**DOI:** 10.1007/s12011-020-02100-3

**Published:** 2020-03-14

**Authors:** Ewelina Król, Monika Okulicz, Justyna Kupsz

**Affiliations:** 1 Institute of Human Nutrition and Dietetics, Poznań University of Life Sciences, ul Wojska Polskiego 31, 60-624 Poznan, Poland; 2 Department of Animal Physiology and Biochemistry, Poznań University of Life Sciences, ul Wołyńska, Poznan, Poland; 3 Department of Physiology, Poznań University of Medical Sciences, ul, ul Święcickiego 6, 61-781 Poznan, Poland

**Keywords:** Taurine, High-fat diet, Iron, Zinc, Copper

## Abstract

Taurine (Tau) is a β-sulphonated amino acid postulated to improve glucose homeostasis in insulin resistance and diabetes. Changes in carbohydrate metabolism are accompanied by oxidative stress, which may disturb the mineral balance. Therefore, the aim of this study was to assess the effect of Tau supplementation on the levels of trace elements in rats fed either a standard (AIN-93M, 4% fat) diet or a modified high-fat diet (30% fat). For 8 weeks, male Wistar rats were fed these diets supplemented with 3% Tau. Taurine supplementation normalized increased serum insulin concentration and insulin resistance index; however, it did not improve serum CRP concentration in high-fat diet fed rats. The high-fat diet supplemented with Tau decreased the renal and splenic Zn levels, but the tissular Fe content did not change. The effect of Tau supplementation on the mineral balance to some extent depended on the fat content in the rats’ diet. The high-fat diet supplemented with Tau decreased the rats’ splenic Zn levels but increased their femur levels. In the group fed the standard diet, Tau reduced the rats’ femur Zn level, whereas their splenic Zn level was comparable. Tau supplementation decreased the renal Cu level and serum ceruloplasmin concentration in the rats fed the standard diet, but this effect was not observed in the rats fed the high-fat diet. In conclusion, supplementary taurine failed to ameliorate disturbances in mineral homeostasis caused by high-fat diet feeding and led to tissular redistribution of Zn and Cu in the rat.

**Electronic supplementary material:**

The online version of this article (10.1007/s12011-020-02100-3) contains supplementary material, which is available to authorized users.

## Introduction

In recent years, the incidence of type 2 diabetes has increased mostly due to environmental factors, i.e. improper dietary behaviours and low physical activity, as well as due to genetic factors. However, it is noteworthy that it takes many years for diabetes to develop and the disease often does not have visible symptoms. It is believed that insulin resistance in target tissues is one of the first signs of carbohydrate metabolism malfunction. Reference publications provide few descriptions of the mechanisms through which a high-fat diet may lead to insulin resistance. The mechanism of insulin resistance (IR) induced by feeding rodents a high-fat diet varies according to the feeding time. At the initial stage of IR, acute lipid overload was observed in tissues. As the IR progressed over time, obesity mediated macrophage-induced proinflammatory actions [1]. The long-term ingestion of a high-fat diet resulted in inflammation in the central nervous system and peripheral tissues (muscle, liver, adipose tissue) [2]. One of them included the ROS production pathway in the liver and adipose tissue. According to Matsuzawa-Nagata et al. [3], in the liver, this process includes the upregulation of genes involved in sterol regulatory element-binding protein 1c–related fatty acid synthesis and peroxisome proliferator–activated receptor *α*–related fatty acid oxidation, while in the adipose tissue, the process includes the downregulation of genes involved in fatty acid synthesis and upregulation of the nicotinamide adenine dinucleotide phosphate (NADPH) oxidase complex. The high-fat diet increased the mitochondrial ROS production in the liver [4]. These processes elevated the tumour necrosis factor-*α* and free fatty acid levels in the plasma and liver [3]. Additionally, few studies describe the relationship between increased oxidative stress, insulin resistance and adipocytokine levels [5, 6].

Oxidative stress is related with insulin resistance and diabetes progression [7]. The biomarkers of plasma oxidative stress were found to be related with the degree of insulin resistance in humans [8]. Oxidative stress is affected by excessive endogenous oxidative species prone to cell damage and influence on the signal pathway [9]. Reactive oxygen species are produced in cells at low physiological levels. The first step in ROS production is the superoxide anion. It can cross the biological membrane via anion channels and react with various site-specific enzymes (extracellular, cytoplasmic and mitochondrial) and the superoxide dismutase (SOD) family, which converts superoxide into molecular oxygen and hydrogen peroxide [10]. Some trace elements are engaged in redox reactions, e.g. Zn, Cu and Fe. Transition metals such as iron (Fe^2+^) and copper (Cu^+^) may increase free radical production and thus exacerbate oxidative stress. For example, Fe^2+^ may cause autoxidation and lead to the generation of O_2_^•−^, whereas interaction with H_2_O_2_ may generate OH**˙** in the Fenton and Haber-Weiss reactions [11]. The transition of Cu(I) and Cu(II) may result in the generation of ROS and it may further lead to the oxidation of protein and other molecules [12, 13]. Although, Zn ions are protected from oxidation through binding to thiol groups [12], this metal is a part of superoxide dismutase (Cu/Zn—SOD). Additionally, zinc is necessary for insulin production, storage and secretion in β cells in the pancreas [14].

Taurine is a β-sulphonated amino acid, which is naturally synthesized from precursors (methionine and cysteine), but the rate of biosynthesis is limited in mammalians [15]. From the nutritional point of view, this compound belongs to conditionally essential amino acids. Taurine depletion in the taurine transporter (TauT) knockout mouse model caused disturbances in glucose metabolism [16]. There were lower plasma taurine levels observed in mice with obesity induced by high-fat feeding and/or genetic manipulation (KKA^y^ mice). Due to higher energy expenditure, taurine supplementation prevented changes induced by obesity [17]. In alloxan-induced diabetes, orally administered taurine decreased elevated glucose and TNF-α, IL-6 levels and diminished renal oxidative stress markers [18]. Therefore, taurine supplementation could be effective in managing insulin resistance, obesity and diabetes. There is no data, whether this intervention can affect trace elements homeostasis in the body.

The aim of this study was to assess the effect of taurine supplementation on insulin resistance; balance of Fe, Zn and Cu; and parameters of oxidative stress in rats fed either control or high-fat diets.

## Material and Methods

### Animals and Diets

Seven-week-old male Wistar rats (*n* = 32) with body weights of 188–248 g were purchased from the Licenced Laboratory Animals Breeding Centre (Poznań, Poland). During the adaptation and the experimental period, the animals were housed at controlled temperature (21 ± 2 °C), humidity (55–60%) and at a 12 h/12 h day/night cycle. After the 7-day adaptation period, the animals were divided into 4 groups with 8 rats in each: C—fed the AIN-93M diet, a healthy control group; Control + Tau—a group fed the AIN-93M diet supplemented with 3% taurine; HF—a group fed a high-fat diet; and HF + TAU—a group fed a high-fat diet supplemented with 3% taurine. The rats were kept individually in semi-metabolic cages. The animals were fed the standard and high-fat diets ad libitum. According to the AIN-93M recommendations [19], the diet was composed of casein (14%), sunflower oil (7%), wheat starch (53.2%), sucrose (10%), potato starch (5%), l-cysteine (0.3%), vitamin mix AIN-93M (1%) and mineral mix AIN-93M (3.5%). The high-fat diets were obtained from the basal AIN-93M diet by replacing wheat starch with fat (up to 30% w/w) as additional fat source in the diet lard was used (Supplementary file, Table 1A). The high fat diet provided app. 50% energy from fat. The diets were prepared once a week and stored at a temperature of 4 °C. Table 1 shows the chemical composition of the experimental diets.

**Table 1:** The chemical composition of diets used in experiment

Ingredient	Experimental diets			
	C	C + TAU	HF	HF + TAU
Protein (%)	12.76 ± 0.30	14.81 ± 0.12	11.37 ± 0.20	13.32 ± 0.09
Fat (%)	4.90 ± 0.04	4.88 ± 0.14	30.27 ± 0.21	30.28 ± 0.44
Carbohydrates (%)	70.79 ± 0.25	68.99 ± 0.21	53.10 ± 0.66	50.50 ± 0.38
Ash (%)	2.91 ± 0.15	2.77 ± 0.17	2.62 ± 0.21	2.77 ± 0.04
Fe (mg/kg)	47.32 ± 3.47	46.52 ± 4.65	47.34 ± 2.54	47.07 ± 3.23
Zn (mg/kg)	37.56 ± 3.53	36.99 ± 2.45	37.48 ± 2.94	37.50 ± 3.13
Cu (mg/kg)	6.28 ± 0.78	6.34 ± 0.99	5.99 ± 0.78	6.01 ± 1.36

*C* standard diet (AIN-93M), *C + TAU* standard diet (AIN-93M) supplemented with 3% taurine, *HF* high-fat diet, *HF + TAU* high-fat diet supplemented with 3% taurine

The rats were fed these diets for 8 weeks. Every day the consumption was assessed, taking the unconsumed amounts remaining in the feeder and tray into account. The body weight gain was measured weekly.

### Reagents

Taurine, tris(2-carboxyethyl)phosphine and 2-chloro-1-methylquinolinium tetrafluoroborate were purchased from Sigma-Aldrich (Saint Louis, MO, USA).

### Data Collection

At the end of the experiment, after 16-h fasting, the rats were anaesthetized with an intraperitoneal thiopental (40 mg/kg b.w.) injection and dissected to collect the blood from the aorta and remove inner organs (liver, kidneys, heart, spleen, pancreas, testes) for biochemical tests. The organs were washed in saline, weighed and stored at − 20 °C until analysis.

### Blood Biochemistry

The blood biochemistry indices were analysed with a COBAS INTEGRA analyser (Roche Diagnostics). The serum glucose concentration was measured with the hexokinase method [20]. The urea concentration was measured with the kinetic method, using urease and glutamine dehydrogenase [21]. The total protein concentration was measured with the colorimetric method, using Cu^+2^ ions [22]. The creatinine concentration was measured with the Jaffe kinetic method, using picric acid [23]. The activities of alanine aminotransferase (ALT) and aspartate aminotransferase (AST) were measured with kinetic methods [23].

The level of hormones in the blood serum was measured with commercial assay kits, i.e. the insulin level—with a rat-specific immunoradiometric (RIA) kit (Linco Reaserch, St. Charles, MO, USA)—and the adiponectin level—with a mouse-specific immunoradiometric kit (Millipore, St. Charles, MO, USA). The concentration of ceruloplasmin in the serum was measured by means of enzyme immunoassay (ADVIA Centaur XP analyser, Siemens Healthcare, Bohemia, NY, USA). The serum glutathione *S*-transferase (GST) and C-reactive protein (CRP) concentrations were measured with rat-specific kits (Cayman Chemical; 1180 East Ellsworth Road, Ann Arbor, Michigan, USA; Wuhan EIAab Science Co., Biopark, Optics Valley, Wuhan, China).

Insulin resistance was characterized with the homeostasis model assessment (HOMA-IR) indices adjusted to the rodent model [24, 25].

### Plasma Homocysteine and Glutathione Concentration Measurements

The blood for total homocysteine (tHcy) and glutathione (GSH) analyses was collected into tubes containing ethylenediaminetetraacetic acid, immediately placed on ice and centrifuged at 800×*g* for 15 min at room temperature. Then, the plasma was stored at − 20 °C. The tHcy and GSH concentrations were measured by means of high-performance liquid chromatography, using the method described by Głowacki and Bald [26, 27]. Briefly, disulphides were reduced with tris(2-carboxyethyl)phosphine, and the newly formed sulphhydryl groups were derivatized with a thiol-specific ultraviolet labelling reagent (2-chloro-1-methylquinolinium tetrafluoroborate). Twenty microlitres of each sample was applied to an Agilent Zorbax SB18 (4.6 × 150 mm; 5 μm) column. Homocysteine and glutathione were eluted under gradient conditions. Solvent A consisted of 0.1 M trichloroacetic acid adjusted to pH 1.65 with NaOH; solvent B was 100% acetonitrile. The HPLC column was equilibrated with 89% solvent A and 11% solvent B. The gradient consisted of 3 min at the equilibration conditions, 10 min to increase solvent B to 35% and 2 min to return to the equilibration conditions. The flow rate was 1.2 ml/min, and the absorbance was monitored at 355 nm. The HPLC was carried out at room temperature. Homocysteine and glutathione were identified according to their retention time and co-chromatography with Hcy and GSH standards. Quantification was based on a comparison of the peak height with the standard calibration curves of Hcy and GSH.

### Microelement Measurements

Diet samples were weighed (2 g) in quartz crucibles, dried at 105 °C for 24 h and subsequently ashed in the muffle furnace with raising temperature to 400 °C. White ash was dissolved in 1 mol/l nitric acid (GR ISO, Merck KGaA, Darmstadt, Germany) and filled up in 50 ml volumetric flasks to the mark by the same acid.

Serum samples were diluted twice with a Triton-X100 solution. Samples of tissues were weighed and digested in 65% (w/w) spectra pure HNO_3_ (Suprapur, Merck KGaA, Darmstadt, Germany) in a Microwave Digestion System (MARS 5, CEM Corp., Matthews NC, USA).

The content of Fe, Zn and Cu in the mineral solutions was measured by means of flame AAS method (AAS- 3 spectrometer, Carl-Zeiss, with BC, Germany). The accuracy of quantitative measurements of minerals was assured by simultaneous analysis of certified reference materials (serum HUMASY CONTROL 2, Randox, Cumlin, UK and Pig Kidney BCR® No. 185, Brussels). The average recovery rates expressed as percentage of estimated values were 102% for Fe, 95% for Zn and 8% for Cu in the serum and 105% for Fe, 98% for Zn and 102% for Cu in tissue mineralisates.

### Statistical Analysis

All the data were expressed as arithmetic means ± standard deviation (SD). The data were analysed statistically with the Statistica for Windows (ver. 13.1, Tulsa, StatSoft PL). The differences between the groups were compared by means of one-way analysis of variance and post-hoc Tukey’s test. The interaction between the factors (the type of diet and taurine supplementation) were compared by means of two-way analysis of variance. The associations between variables were checked with the Pearson’s correlation coefficient. The significance level was set at *α* = 0.05.

## Results

The average intake of the high-fat diet was lower than the intake of the AIN-93M diet (*p* < 0.001) (Table 2). Thus, the groups fed those diets consumed lower amounts of minerals (Fe, Zn and Cu) (*p* < 0.001) than the rats fed the standard diets. The taurine supplementation did not influence these indices (*p* > 0.05).

**Table 2:** Daily diet and Fe, Zn and Cu intake in rats

Parameter	Experimental group				ANOVA *p* value		
	C	C + TAU	HF	HF + TAU	Diet	TAU	Diet × TAU interaction
Diet (g/day)	20.17 ± 1.02^b^	20.34 ± 1.36^b^	16.89 ± 0.61^a^	17.02 ± 1.22 ^a^	< 0.001	NS	NS
Fe (g/day)	0.73 ± 0.04^b^	0.72 ± 0.05^b^	0.61 ± 0.02^a^	0.61 ± 0.05 ^a^	< 0.001	NS	NS
Zn (g/day)	0.62 ± 0.03^b^	0.63 ± 0.05^b^	0.52 ± 0.02^a^	0.53 ± 0.04 ^a^	< 0.001	NS	NS
Cu (g/day)	0.13 ± 0.00^b^	0.13 ± 0.00^b^	0.11 ± 0.00^a^	0.11 ± 0.00 ^a^	< 0.001	NS	NS

Values are presented as means ± standard deviation; values in the row with different superscripts differ significantly (*p* < 0.05)

*C* rats fed standard diet (AIN-93M) control group, *C + TAU* rats fed standard diet (AIN-93M) supplemented with 3% taurine, *HF* rats fed high-fat diet, *HF + TAU* rats fed high-fat diet supplemented with 3% taurine, *NS* statistically non-significant difference

Table 3 shows the effect of the high-fat diet and taurine supplementation on the blood biochemical indices. The serum glucose concentration was comparable in all the experimental groups (*p* > 0.05). In comparison with the group fed the AIN-93M diets, the insulin concentration and HOMA-IR index of the rats fed the high-fat diet increased significantly, i.e. by 60% and two times, respectively (*p* < 0.05). The taurine supplementation in the high-fat diet normalized both indices to a certain extent. The serum adiponectin level was comparable in all the groups. Increased ALT activity was observed in the rats fed the high-fat diet. The taurine supplementation normalized its level. Neither the high-fat diet nor taurine affected other liver and kidney toxicity indices, such as the AST activity, urea and creatinine concentration (*p* > 0.05).

**Table 3:** Effect of taurine supplementation on biochemical markers in rats (mean ± SD)

Parameter	Experimental group				ANOVA *p* value		
	C	C + TAU	HF	HF + TAU	Diet	TAU	Diet × TAU interaction
Glucose (mmol/L)	7.75 ± 1.40	7.67 ± 1.67	9.51 ± 1.08	9.13 ± 1.68	NS	NS	NS
Insulin (mIU/L)	8.77 ± 1.76^a^	9.33 ± 1.74^a^	12.84 ± 3.07^b^	9.84 ± 2.98^ab^	< 0.05	< 0.05	NS
HOMA-IR	2.66 ± 0.65^a^	2.93 ± 0.74^a^	4.66 ± 0.89^b^	3.88 ± 1.36^ab^	< 0.05	< 0.05	NS
Serum adiponectin (μg/L)	2.31 ± 0.57	2.83 ± 0.88	2.11 ± 0.74	2.72 ± 0.92	NS	NS	NS
Homocysteine (μmol/L)	5.41 ± 1.76	5.35 ± 0.89	6.41 ± 2.25	5.02 ± 0.72	NS	NS	NS
ALT activity (U/L)	16.75 ± 2.05^a^	16.60 ± 1.67^a^	23.88 ± 5.77^b^	18.57 ± 3.41^ab^	< 0.01	NS	NS
AST activity (U/L)	73.87 ± 12.90	74.80 ± 10.63	65.51 ± 9.58	68.71 ± 11.74	NS	NS	NS
Urea (mmol/L)	4.23 ± 0.61	4.55 ± 0.53	4.00 ± 0.45	4.61 ± 0.95	NS	NS	NS
Creatinine (μmol/L)	45.08 ± 3.54	44.20 ± 5.30	44.20 ± 4.42	45.97 ± 3.54	NS	NS	NS

Values are presented as means ± standard deviation; values in the row with different superscripts differ significantly (*p* < 0.05)

*C* rats fed standard diet (AIN-93M) control group, *C + TAU* rats fed standard diet (AIN-93M) supplemented with 3% taurine, *HF* rats fed high-fat diet, *HF + TAU* rats fed high-fat diet supplemented with 3% taurine, *NS* statistically non-significant difference

Table 4 shows the influence of dietary fat and taurine supplementation on zinc tissular levels. The high-fat diet significantly decreased the hepatic (*p* < 0.001), renal (*p* < 0.001), splenic (*p* < 0.01) and femoral (*p* < 0.001) zinc levels. The taurine supplementation decreased the zinc content in the kidney in both supplemented group (*p* < 0.001) and in the spleen in high-fat diet fed group (*p* < 0.05). Moreover, there were interaction effects observed. When taurine was added to the high-fat diet, it decreased the splenic zinc level (*p* < 0.05) but increased the femoral level (*p* < 0.001). In rats fed standard diets, the effects were opposite.

**Table 4:** Effect of taurine supplementation on tissular zinc levels in rats (mean ± SD)

Parameter	Experimental group				ANOVA *p* value		
	C	C + TAU	HF	HF + TAU	Diet	TAU	Diet × TAU interaction
Serum Zn (mmol/L)	22.51 ± 3.71	20.29 ± 1.45	20.18 ± 2.24	18.65 ± 2.88	NS	NS	NS
Hepatic Zn (μg/g d.m.)	117.11 ± 16.78^b^	116.85 ± 14.18^b^	92.40 ± 9.32^a^	91.61 ± 6.93^a^	*p* < 0.001	NS	NS
Renal Zn (μg/g d.m.)	94.63 ± 4.06^c^	82.84.63 ± 12.08^bc^	80.02 ± 2.91^b^	60.48 ± 7.54^a^	*p* < 0.001	*p* < 0.01	NS
Splenic Zn (μg/g d.m.)	86.68 ± 17.41^b^	96.28 ± 14.05^b^	81.02 ± 20.82^b^	63.88 ± 12.73^a^	*p* < 0.01	NS	*p* < 0.05
Cardiac Zn (μg/g d.m.)	51.76 ± 13.41	43.43 ± 10.76	44.81 ± 9.25	41.03 ± 6.73	NS	NS	NS
Femur Zn (μg/g d.m.)	218.96 ± 24.39^c^	172.80 ± 12.08^b^	123.07 ± 17.51^a^	144.16 ± 14.57^ab^	*p* < 0.001	NS	*p* < 0.001

Values are presented as means ± standard deviation; values in the row with different superscripts differ significantly (*p* < 0.05)

*C* rats fed standard diet (AIN-93M) control group, *C + TAU* rats fed standard diet (AIN-93M) supplemented with 3% taurine, *HF* rats fed high-fat diet, *HF + TAU* rats fed high-fat diet supplemented with 3% taurine, *NS* statistically non-significant difference

Changes in copper metabolism were significantly marked either by high-fat feeding or taurine supplementation (Table 5). The high-fat diet significantly decreased the hepatic (*p* < 0.001) and renal (*p* < 0.001) Cu content in comparison with the groups fed the standard AIN-93M diets.

**Table 5:** Effect of taurine supplementation on serum and tissular Cu concentration in rats (mean ± SD)

Parameter	Experimental group				ANOVA *p* value		
	C	C + TAU	HF	HF + TAU	Diet	TAU	Diet × TAU interaction
Serum Cu (mmol/L)	16.13 ± 1.76^b^	13.48 ± 1.14^ab^	13.48 ± 0.68^ab^	12.09 ± 4.51^a^	NS	NS	NS
Hepatic Cu (μg/g d.m.)	18.47 ± 2.86^b^	17.71 ± 5.14^b^	12.14 ± 0.85^a^	12.40 ± 1.66^a^	< 0.0001	NS	NS
Renal Cu (μg/g d.m)	39.00 ± 9.33^b^	22.38 ± 5.46^a^	18.97 ± 4.02^a^	16.14 ± 3.23^a^	< 0.001	< 0.0001	< 0.01
Splenic Cu (μg/g d.m.)	8.14 ± 2.82	8.91 ± 3.65	8.00 ± 4.74	9.26 ± 3.83	NS	NS	NS
Cardiac Cu (μg/g d.m.)	20.47 ± 2.45^b^	18.96 ± 6.34^ab^	15.93 ± 2.81^ab^	13.96 ± 1.88^a^	< 0.05	NS	NS
Femur Cu (μg/g d.m.)	2.52 ± 0.76	2.71 ± 0.33	2.19 ± 0.41	2.45 ± 0.26	NS	NS	NS

Values are presented as means ± standard deviation; values in the row with different superscripts differ significantly (*p* < 0.05)

*C* rats fed standard diet (AIN-93M) control group, *C + TAU* rats fed standard diet (AIN-93M) supplemented with 3% taurine, *HF* rats fed high-fat diet, *HF + TAU* rats fed high-fat diet supplemented with 3% taurine, *NS* statistically non-significant difference

The addition of taurine to the diet decreased the kidney Cu levels (*p* < 0.001) and serum ceruloplasmin level (*p* < 0.05). The interaction effects between the type of diet and taurine supplementation were observed for the renal Cu level (*p* < 0.01) and serum ceruloplasmin concentration (*p* < 0.001). The taurine supplementation decreased the renal Cu level and increased serum ceruloplasmin concentration of the rats fed the standard diet, but these effects were not observed in the rats fed the high-fat diet.

Neither high-fat diet nor dietary taurine influences on iron balance in the rat (Table 6).

**Table 6:** Effect of taurine supplementation on tissular iron levels in rats (mean ± SD)

Parameter	Experimental group				ANOVA *p* value		
	C	C + TAU	HF	HF + TAU	Diet	TAU	Diet × TAU interaction
Serum Fe (mmol/L)	26.54 ± 4.11	25.98 ± 2.34	28.13 ± 2.26	26.56 ± 3.03	NS	NS	NS
Hepatic Fe (μg/g d.m.)	360.08 ± 67.60	423.04 ± 20.37	400.66 ± 68.38	449.01 ± 68.64	NS	NS	NS
Renal Fe (μg/g d.m.)	263.01 ± 35.60	223.52 ± 30.97	253.00 ± 52.72	209.50 ± 36.88	NS	NS	NS
Splenic Fe (μg/g d.m.)	2949.51 ± 572.87	3211.67 ± 453.38	3254.94 ± 714.10	3454.22 ± 360.15	NS	NS	NS
Cardiac Fe (μg/g d.m.)	332.62 ± 34.19	326.18 ± 45.08	343.58 ± 71.53	302.35 ± 45.06	NS	NS	NS
Femur Fe (μg/g d.m.)	80.24 ± 9.50	78.89 ± 17.40	75.27 ± 13.16	69.76 ± 10.17	NS	NS	NS

Values are presented as means ± standard deviation

*C* rats fed standard diet (AIN-93M) control group, *C + TAU* rats fed standard diet (AIN-93M) supplemented with 3% taurine, *HF* rats fed high-fat diet, *HF + TAU* rats fed high-fat diet supplemented with 3% taurine, *NS* statistically non-significant difference

Table 7 shows the effects of the high-fat diet and taurine supplementation on the serum antioxidative indices and C-reactive protein levels. Neither the high-fat diet nor taurine affected the rats’ serum superoxide dismutase, glutathione levels and GST. The high-fat diet significantly increased the serum C-reactive protein level (*p* < 0.05), but the taurine supplementation did not normalize this parameter.

**Table 7:** Effect of taurine supplementation on CRP and serum antioxidant enzymes in rats (mean ± SD)

Parameter	Experimental group				ANOVA *p* value		
	C	C + TAU	HF	HF + TAU	Diet	TAU	Diet × TAU interaction
Ceruloplasmin (mg/L)	861.09 ± 256.79	1314.63 ± 175.45	947.26 ± 197.82	935.45 ± 151.07	NS	< 0.05	< 0.001
GSH (μmol/L)	32.20 ± 2.64	31.36 ± 3.96	32.76 ± 2.35	38.83 ± 11.20	NS	NS	NS
Cu/Zn SOD (μg/L)	15.45 ± 2.09	16.45 ± 3.71	13.29 ± 4.30	16.32 ± 3.63	NS	NS	NS
GST (μmol/min/L)	4.02 ± 0.45	2.98 ± 1.19	2.98 ± 1.43	3.68 ± 0.73	NS	NS	NS
CRP (mg/L)	0.102 ± 0.048^a^	0.098 ± 0.029^a^	0.182 ± 0.074^b^	0.187 ± 0.056^b^	< 0.05	NS	NS

Values are presented as means ± standard deviation; values in the row with different superscripts differ significantly (*p* < 0.05)

*C* rats fed standard diet (AIN-93M) control group, *C + TAU* rats fed standard diet (AIN-93M) supplemented with 3% taurine, *HF* rats fed high-fat diet, *HF + TAU* rats fed high-fat diet supplemented with 3% taurine, *Cu/Zn SOD* superoxide dismutase, *GSH* glutathione, *GST* glutathione *S*-transferase, *CRP* C-reactive protein, *NS* statistically non-significant difference

Table 8 shows the significant correlation coefficients between serum biochemical indices and tissular mineral levels in rats. The positive associations were found between femur Cu level and serum ceruloplasmin concentration (*r* = 0.520) and HOMA-IR (*r* = 0.523). The serum glucose inversely correlated with hepatic and femur Zn level (*r* = − 0.370 and *r* = − 0.360, respectively) and cardiac Cu (*r* = − 0.427), while HOMA-IR with femur Zn (*r* = − 0.420) and hepatic Cu level (*r* = − 0.420). There were inverse association between splenic Zn and serum glutathione (*r* = − 0.411).

**Table 8:** Significant correlation coefficients between tissular mineral levels and biochemical indices in all rats

Correlation	*R*^2^	*p*
Hepatic Zn/serum glucose	− 0.370	0.047
Femur Zn/serum glucose	− 0.360	0.046
Femur Zn/ HOMA-IR	− 0.420	0.023
Splenic Zn/serum glutathione	− 0.411	0.026
Hepatic Cu/HOMA-IR	− 0.420	0.046
Cardiac Cu/serum glucose	− 0.427	0.021
Femur Cu/serum ceruloplasmin	0.520	0.009
Femur Cu/HOMA-IR	0.523	0.009

*HOMA-IR* Homeostasis Model Assessment for Insulin Resistance

## Discussion

One of the aims of this study was to examine the effect of taurine supplementation on glucose metabolism. In our study, taurine did not directly influence the serum glucose concentration, but to some extent, it normalized the serum insulin and insulin resistance index HOMA-IR in the rats fed the high-fat diet. Kim et al. [28] studied the role of taurine in the modulation of insulin sensitivity and insulin secretion in Otsuka Long-Evans Tokushima fatty (OLETF) rats. The study on this long-term diabetes model showed that taurine had reduced the blood glucose levels, improved OGTT measurements and reduced insulin resistance without affecting the β cell function or pancreatic islet mass. Other studies showed that taurine protected islets and hepatocytes against lipotoxicity induced by the infusion of oleate or fatty acids [29, 30].

Borck et al. [31] noticed that 5% taurine solution given to obese mice with drinking water for 12 months decreased TAG and improved glucose tolerance and insulin sensitivity. In another study conducted by this team, 2.5% TAU solution given to hypothalamic obese rats for a shorter period (130 days) did not affect the fasting glucose level, but decreased the insulin and TAG concentration [32]. These results support the hypothesis that taurine supplementation may prevent diabetes in obese subjects. The mechanisms by which taurine improves β cell function are linked to its role in the regulation of glucose transport, K_ATP_ channel operation, Ca^2+^ handling, mitochondrial metabolism and granule exocytosis in β cells [33]. Increased taurine concentration in pancreatic islet due to supplementation and the inhibitory autocrine action of insulin may prevent the insulin hypersecretion and maintain a normal β cell mass [34]. Additionally, taurine may affect glucose homeostasis by insulin clearance, increasing the expression of zinc metalloprotease—insulin degrading enzyme (IDE) in the liver of rodents [35].

The adipose tissue plays an important role in insulin resistance as it secretes adipocytokines such as adiponectin. Adiponectin is related to glucose, lipid metabolism and oxidative stress. De Rosa et al. [36] found that whole adiponectin oligomers were inversely correlated with BMI, some biochemical indices (glucose, insulin, TAG), insulin resistance and visceral fat accumulation. Although the serum taurine concentration was not measured in our study, earlier studies revealed a positive correlation between the serum adiponectin and serum taurine concentrations in obese animals supplemented with taurine [37]. This fact supports the hypothesis that taurine improves insulin resistance. Other authors also studied the influence of taurine supplementation on adipokines levels. Taurine administered to chronic alcohol-fed rats prevented the decline of the serum adiponectin level [38]. However, in our study, taurine did not significantly affect the serum adiponectin levels of the rats in the control and high-fat diet groups. There was only a positive trend observed. It may have been caused by the relatively short time of supplementation.

Our supplementary study was not a dose-response study; thus, we used only one dose of taurine. In mammals, the kidneys are responsible for taurine homeostasis. Although the reabsorption of amino acids by the kidney is highly efficient, in case of taurine, excretion rate depends on dietary intake. The higher the dietary taurine intake is, the higher the urinary excretion of this amino acid [39].

As mentioned above, a high-fat diet is accompanied by obesity, insulin resistance and oxidative stress. These changes led to fluctuations in the expression of antioxidant defence enzymes, such as superoxide dismutases, glutathione and malondialdehyde. Some studies revealed negative relations between the plasma EC-SOD levels and glucose, as well as HOMA-IR and BMI, and positive relations between adiponectin in type 2 diabetics [40].

Taurine is indirect antioxidant. It regulates mitochondrial protein synthesis, thus enhances electron transport chain activity and protects the mitochondria against excessive superoxide generation [41]. As earlier research findings indicated the antioxidative potential of taurine, we studied the effects of the diet and taurine on some antioxidant enzymes in the rats’ serum. Although the serum does not give full insight into oxidative processes occurring in organs, some researchers found significant correlations between the blood and tissular level of some biomarkers [42]. In our study, the high-fat diet slightly decreased the serum SOD and GST levels but increased the proinflammatory marker (C-reactive protein). However, taurine did not improve these markers significantly. There were no changes in other oxidative stress parameters due to the relatively short time of supplementation or the rats’ adaptive mechanism to the high-fat diet.

As copper and zinc are counterparts of Cu/Zn superoxide dismutase, we examined the effect of fat and taurine in the rats’ diet on the homeostasis of these metals. The higher content of fat in the diet can change the mineral status. Derangements in the status of trace elements might be associated with insulin resistance. Special attention is paid to copper, zinc and iron. As copper is capable of receiving and donating electrons, it participates in many catalytic reactions. This metal can act as an anti- or pro-oxidant; it can scavenge or neutralize free radicals or promote free radicals damage, respectively. Apart from the antioxidant defence, Cu-binding enzymes or proteins are engaged in many biochemical processes, e.g. iron mobilisation, Cu transport, blood clotting, electron transfer and metal detoxification [43]. On the other hand, zinc plays a role in insulin signalling. Maret [44] hypothesized that low Zn content in the liver could be associated with hepatic insulin resistance. In our study the high-fat diet decreased the hepatic and renal Zn and Cu content as well as the femur Zn and serum Cu levels. The fat content in the diet did not affect the serum and tissular Fe levels. These results are similar to other research findings, where diminished hepatic [45, 46] and renal [47] Zn concentrations were observed. Similarly, the renal Cu content decreased in the rats fed a high-fat diet [48], those fed a high-fat/high fructose diet [47] and in the rats with non-alcoholic fatty liver disease (NAFLD) [46]. Other authors also observed reduced serum Cu concentration [45]. The high-fat diet did not affect the Fe content in laboratory animals’ liver [45–47], kidney [47, 48] and serum [45, 46, 48]. The changes in the mineral status may have been caused by the fact that the rats fed high-fat diets had lower absorption and/or lower intake of these minerals. Some authors suggested that changes of mineral homeostasis precede obesity associated metabolic disturbances [45]. In our study, changes in hepatic and renal Cu and Zn contents were associated with hyperinsulinemia, insulin resistance and C-reactive protein concentration.

To the best of our knowledge, this is the first study on the influence of dietary taurine on the homeostasis of trace elements in animals with insulin resistance induced by a high-fat diet. We investigated whether a moderate dose of taurine (ca. 1.2 g/kg b.m.) affected the tissular zinc and copper levels of the rats fed the standard and high-fat diets with adequate zinc and copper levels. The 8-week taurine supplementation changed the tissular zinc and copper homeostasis. Taurine in the standard diet reduced the femoral Zn level and the Cu content in the kidney. These changes were accompanied by higher serum ceruloplasmin levels (the copper transport protein) in the rats fed the standard diet supplemented with taurine. On the other hand, taurine supplementation in the high-fat diet decreased the renal and splenic Zn contents but slightly increased its femoral level. The metabolic consequences of these changes are difficult to assess because at the same time, an increase in the content of this element in the femur was noted. Zinc deficiency is less likely because in rats fed diets depleted with zinc decreased serum, liver and femur Zn concentrations and decreased glutathione *S*-transferase activity were observed [49–51]. Moreover, zinc depletion led to diminished hepatic copper content [50].

There are hardly any data on the influence of taurine on zinc and copper metabolism. Researchers observed the positive effect of taurine supplementation on rats intoxicated with heavy metals such as copper and zinc. Hwang et al. [52] found that dietary taurine supplementation decreased the Cu content in the serum, liver and kidney but increased the level of Cu excreted with the faeces of animals fed diets with copper excess (up to 600 ppm). The authors suggested that this amino acid may reduce the overall bioavailability of copper or the intracellular availability of absorbed copper. In another study, these researchers studied the role of taurine in reducing zinc-induced toxicity in rats. Taurine to some extent reduced the toxicity resulting from high doses of zinc (up to 600 ppm). The 5% dose of taurine decreased the elevated plasma, liver and kidney zinc levels but increased the levels of zinc excreted with faeces [53]. The taurine supplementation had positive antioxidative effect on rats intoxicated with these metals because their oxidative stress markers were elevated.

On the other hand, Choi et al. [54] observed that taurine supplemented at a dose of 1.5% of the diet did not affect the plasma zinc level or urinary extraction of this element in alcohol-consuming rats. In other study, short-term (a single dose of 200 mg/kg/day for 7 days) taurine supplementation did not affect hepatic zinc and copper content in young and old rats [55].

However, Glover and Hogstrand investigated the influence of amino acids on in vivo intestinal zinc absorption in freshwater rainbow trout [56]. The authors found that higher doses of taurine (100 mmol/L) increased the subepithelial and decreased the post-intestinal Zn(II) content. Additionally, the Zn(II) accumulation rate decreased in the blood and carcass exposed to the same taurine concentration.

The mechanism by which taurine interacts with Zn has not been explained. In vitro taurine showed chelating activity against copper and zinc, but not iron. The ability was weaker than other sulphur-containing amino acids [57]. On the other hand, according to Harraki et al. [58], the structure of taurine molecule is unlikely to indicate the possibility of zinc chelation. The authors found that taurine is unable to displace zinc from albumin [58]. As many tissues (e.g. retina) are abundant in taurine and zinc, presumably they affect each other. Marquez et al. [59] found that zinc modulated taurine transport in a dose-dependent fashion, acting directly on the transporter or forming taurine-zinc complexes in rats’ retinal cell membranes. The mechanism of this interaction is rather synergistic. It is very likely that taurine may also affect zinc transporters because both of them are present under physiological conditions. Another explanation of this interaction may be the fact that taurine and calcium cooperate in the mobility of phospholipids and fluidity of membranes [60]. Therefore, the influence of taurine on Zn homeostasis may also be a consequence of changes in calcium metabolism. However, this issue needs further investigations. Data on the influence of supplementary taurine on metallothionein levels are also scant. In Sochor et al. study [61], in young rats, metallothionein contents in serum and erythrocytes increased with increasing level of supplementary taurine.

As mentioned earlier, the effect of taurine on mineral metabolism in insulin resistance or diabetes has not been studied, but Zhou et al. [62] examined how taurine derivate tauroursodeoxycholic acid (TUDCA) administered intraperitoneally to type 1 diabetic mice at a dose of 250 mg/kg twice a day for 2 months affected their copper and zinc metabolism. The authors hypothesized that this chaperon might improve the Zn and Cu imbalance in diabetes through the antioxidative effect. TUDCA only normalized the serum Zn level in the diabetic TUDCA group. This compound did not improve the tissular (hepatic, cardiac, splenic, renal and muscle) Zn and Cu imbalances.

The study was limited by the fact that the level of oxidative stress markers was examined only in the serum, without the tissues. Neither the serum taurine level nor the amount of taurine excreted in the urine was measured.

## Conclusions

Our study confirmed that 8-week taurine supplementation to some extent normalized insulin resistance in the rats fed the high-fat diet. This intervention was not able to correct disturbances in mineral homeostasis caused by high-fat diet feeding in the rat. The effect of dietary taurine on tissular mineral levels to some extent depends on the fat content in the diet. It seems that these changes might result from the influence of taurine on the absorption of these elements and to some extent from the antioxidative action, but the mechanism of this interaction requires further investigations.

## Electronic supplementary material

ESM 1 (DOCX 63 kb)
