# Supplementary material for: The Influence of Taurine Supplementation on Serum and Tissular Fe, Zn and Cu Levels in Normal and Diet-Induced Insulin-Resistant Rats
Source: Biol Trace Elem Res. 2020 Mar 14;198(2):592–601. doi: 10.1007/s12011-020-02100-3 (PMC7561555; doi:10.1007/s12011-020-02100-3)
Supplement: Supplementary file 1 [file 12011_2020_2100_MOESM1_ESM.docx]

Table 1. Composition of diets used in experiment (g/kg diet)

Ingredient	C	C+Tau	HF	HF+Tau
Casein	140	140	140	140
Sunflower oil	50	50	50	50
Wheat starch	613.2	583.2	363.2	333.2
Sucrose	100	100	100	100
Potato starch	50	50	50	50
Mineral mix	35	35	35	35
Vitamin mix	10	10	10	10
L-cysteine	1.8	1.8	1.8	1.8
Lard	0	0	250	250
Taurine	0	30	0	30

Abbreviations: C- standard diet (AIN-93M); C+TAU- standard diet (AIN-93M) supplemented with 3% taurine; HF- high-fat diet; HF+TAU - high-fat diet supplemented with 3% taurine
